# A single inhalation of vapor from dried toad secretion containing 5-methoxy-*N*,*N*-dimethyltryptamine (5-MeO-DMT) in a naturalistic setting is related to sustained enhancement of satisfaction with life, mindfulness-related capacities, and a decrement of psychopathological symptoms

**DOI:** 10.1007/s00213-019-05236-w

**Published:** 2019-04-13

**Authors:** M. V. Uthaug, R. Lancelotta, K. van Oorsouw, K. P. C. Kuypers, N. Mason, J. Rak, A. Šuláková, R. Jurok, M. Maryška, M. Kuchař, T. Páleníček, J. Riba, J. G. Ramaekers

**Affiliations:** 1 Department of Neuropsychology and Psychopharmacology, Faculty of Psychology and Neuroscience, Maastricht University, Maastricht, The Netherlands; 2 Innate Path, Lakewood, CO USA; 3 Department of Clinical Sciences, Faculty of Psychology and Neuroscience, Maastricht University, Maastricht, The Netherlands; 4 National Institute of Mental Health, Prague, Czech Republic; 5 1st Faculty of Medicine, Charles University, Prague, Czech Republic; 6 Forensic Laboratory of Biologically Active Substances, Department of Chemistry of Natural Compounds, University of Chemistry and Technology, Prague, Czech Republic; 7 Department of Organic Chemistry, University of Chemistry and Technology, Prague, Czech Republic; 8 3rd Faculty of Medicine, Charles University in Prague, Ruská 87, 10000 Prague 10, Czech Republic

**Keywords:** *Bufo alvarius*, Field study, Cognition, Affect, Altered states of consciousness, Satisfaction with life, Psychedelic, 5-MeO-DMT

## Abstract

**Background:**

5-methoxy-N,N-dimethyltryptamine (hereinafter referred to as 5-MeO-DMT) is a psychedelic substance found in the secretion from the parotoid glands of the *Bufo alvarius* toad. Inhalation of vapor from toad secretion containing 5-MeO-DMT has become popular in naturalistic settings as a treatment of mental health problems or as a means for spiritual exploration. However, knowledge of the effects of 5-MeO-DMT in humans is limited.

**Aims:**

The first objective of this study was to assess sub-acute and long-term effects of inhaling vapor from dried toad secretion containing 5-MeO-DMT on affect and cognition. The second objective was to assess whether any changes were associated with the psychedelic experience.

**Methods:**

Assessments at baseline, within 24 h and 4 weeks following intake, were made in 42 individuals who inhaled vapor from dried toad secretion at several European locations.

**Results:**

Relative to baseline, ratings of satisfaction with life and convergent thinking significantly increased right after intake and were maintained at follow-up 4 weeks later. Ratings of mindfulness also increased over time and reached statistical significance at 4 weeks. Ratings of depression, anxiety, and stress decreased after the session, and reached significance at 4 weeks. Participants that experienced high levels of ego dissolution or oceanic boundlessness during the session displayed higher ratings of satisfaction with life and lower ratings of depression and stress.

**Conclusion:**

A single inhalation of vapor from dried toad secretion containing 5-MeO-DMT produces sub-acute and long-term changes in affect and cognition in volunteers. These results warrant exploratory research into therapeutic applications of 5-MeO-DMT.

**Electronic supplementary material:**

The online version of this article (10.1007/s00213-019-05236-w) contains supplementary material, which is available to authorized users.

## Introduction

5-methoxy-N,N-dimethyltryptamine (5-MeO-DMT) is a potent, fast-acting, psychedelic substance, which acts as a serotonin 5-HT-1-A/5-HT-2A/C receptor agonist (Krebs-Thomson et al. 2006; Ray 2010; Shen et al. 2010). The psychedelic substance 5-MeO-DMT was initially isolated from the bark of the plant *Dictyoloma incanescens* (Pachter et al. 1959) and has also been found in the milky-white secretion produced in the skin and the parotoid glands of the *Bufo alvarius* toad, also known by the scientific name “*Incilius alvarius*” and more commonly referred to as the “Colorado river toad” or “Sonoran desert toad.” Besides 5-MeO-DMT, the secretion of *Bufo alvarius* also contains 5-hydroxy-*N*,*N*-dimethyltryptamine, the closely related O-dimethylated analog of 5-MeO-DMT, commonly known as bufotenine. Moreover, the toad secretion also contains several cardioactive agents like bufagins, various catecholamines such as epinephrine and norepinephrine, and non-cardioactive sterols such as cholesterol, provitamin D, gamma sitosteral and ergosterol (Chen and Kovaříková 1967). Since a toad yields about 0.25–0.50 g of dried secretion from a “milking session,” with a 5-MeO-DMT content of up to 15% (Erspamer et al. 1967), a single toad may generate 75 mg of 5-MeO-DMT in its secretion that, when smoked, is already psychoactive in humans at doses as low as 3–5 mg (Weil and Davis 1994). Users have reported that the psychedelic effects of 5-MeO-DMT are more potent and more intense as compared with other psychedelics (Barsuglia et al. 2017, 2018; Davis et al. 2018). A *s*ingle inhalation of vaporized dried toad secretion produces a psychedelic experience within 15 s (Weil and Davis 1994) and may last up to 20–40 min (Ott 2001).

Some reports suggest that the secretion of the *Bufo alvarius* toad may have been used historically by indigenous peoples in the southwestern territory of the USA and northern Mexico (Weil and Davis 1994). However, the purpose of these ancient practices has not been fully clarified, and evidence of use as a medicine is scarce. Therefore, it cannot be ruled out that the present use of toad secretion is of more recent origin.

A recent survey among users of 5-MeO-DMT (toad, synthetic, or plant derived) indicated that most respondents used 5-MeO-DMT for spiritual exploration and reported mystical-type experiences of moderate-to-high intensity (Davis et al. 2018). Interestingly, those respondents who reported a psychiatric disorder mentioned that 5-MeO-DMT had helped them reduce their symptoms of anxiety, depression, and post-traumatic stress, or effectively deal with alcoholism and drug abuse (Davis et al. 2018; Davis et al. 2019). The potential mental health benefits of 5-MeO-DMT have not been studied in humans but might be similar with those that have been reported for ayahuasca, an Amazonian plant preparation that contains the closely related psychedelic compound, *N*,*N*-dimethyltryptamine (DMT). The potential of ayahuasca to rapidly and persistently reduce symptoms of depression has been shown in a range of open-label studies (De Lima et al. 2011; Sanches et al. 2016), and was recently confirmed in a randomized placebo-controlled clinical trial in patients suffering from treatment-resistant depression (Palhano-Fontes et al. 2018). Moreover, in vitro studies have recently provided evidence of anti-inflammatory, neuroregenerative, and anti-addictive effects induced by 5-MeO-DMT (Dakic et al. 2017; Szabo et al. 2014). Overall, these findings support a potential role for 5-MeO-DMT as a therapeutic tool.

The present study was conducted to obtain initial data on the effects of 5-MeO-DMT on mental health–related functions such as affect and cognition. We visited several locations in three European countries, where people would inhale vapor from dried toad secretion containing 5-MeO-DMT. Our primary objective was to assess whether inhaling the vapor produces any sub-acute and/or long-lasting improvements in measures of affect and cognition. We expected that symptoms of affect, such as depression, anxiety, stress, and somatization reported by participants at baseline would be ameliorated by the use of the vapor from dried toad secretion containing 5-MeO-DMT through the means of inhalation, and that measures of cognition, such as creative thinking- and mindfulness-related capacities, would improve. We also expected that such changes would persist over time and would still be measurable at 4 weeks after intake. As a secondary objective, we aimed to determine whether the sub-acute and long-term effects of inhaling the vapor from the 5-MeO-DMT-rich toad secretion mental health measures were related to certain aspects of the acute psychedelic state, such as ego dissolution and other subjective experiences that characterize the altered state of consciousness. The rationale for studying these associations is based on the results reported in other studies that have demonstrated a direct association between the beneficial effects of psychedelic tryptamines such as psilocybin (Garcia-Romeu et al. 2014; Roseman et al. 2017), the DMT-containing brew ayahuasca (Uthaug et al. 2018), and the ability of these drugs to induce mystical experiences and ego dissolution during the acute psychedelic phase.

## Methods

### Participants

We recruited our sample from three different countries in Europe (Czech Republic (*N* = 25), Spain (*N* = 10), and The Netherlands (*N* = 7)). In all three locations, the participants inhaled vapor from dried toad secretion containing 5-MeO-DMT. The participants were invited to enter the study on site. Overall, 75 participants agreed to participate in the study at baseline. Of those, 42 participants completed the test battery both before inhalation of the vapor from dried toad secretion containing 5-MeO-DMT (baseline) and within 24 h, while only 24 completed the test battery at the third and final assessment 4 weeks after intake.

Most participants were from Europe (*n* = 34 [81%]) while the rest of the participants were from Asia (*n* = 2 [4.8%]), Australia (n = 2 [4.8%]), South America (*n* = 3 [7.1%]), and North America (*n* = 1 [2.4%]). Sixty percent of the sample were males and 40% females. The mean age for the entire group was 38 years (SE = 0.80).

Furthermore, their motivation to inhale vapor from dried toad secretion containing 5-MeO-DMT included increasing self-understanding (*N* = 18 [42.9%]), solving personal problems (*N* = 5 [11.9%]), the combination of both (*N* = 1 [4.2%]), and other (*N* = 18 [42.9%]) reasons. A listing of these “other” motivations or reasons to inhale vapor from dried toad secretion containing 5-MeO-DMT is given in Table 1. In regard to education level, most participants had completed high school (*N* = 14 [33.3%]), a similar percentage had obtained a bachelor’s degree (*N* = 13 [31%]), a Master’s degree (*N* = 11 [26.2%]), and a lower proportion held a Ph.D. (*N* = 3 [7.1%]). One participant had only completed elementary school (*N* = 1 [2.4%]). In total, 15 participants reported having had no previous experience with 5-MeO-DMT (whether this was a toad, synthetic, or plant was unspecified), while the majority (92.9%) had experience with other psychedelics (e.g., LSD, psilocybin, etc.). In regard to mental health status as per self-reports, 32 (76.2%) of the participants reported having no mental health disorder, 1 (2.4%) participant reported having depression, 1 (2.4%) participant reported having a personality disorder, and 1 (2.4%) participant reporting having another mental health disorder not included on the administered list. Furthermore, 4 (9.4%) participants reported having anxiety, while 3 (7.1%) participants reported addiction. As per the nature of this observational study, participants who attended the session to inhale vapor from dried toad secretion containing 5-MeO-DMT were included in the study if they met the inclusion criteria (fluent in either Spanish, Czech, Dutch, or English, were over 18 years of age, and gave their written informed consent). The study was approved by the Ethical Review Committee for Psychology and Neuroscience (ERCPN), from Maastricht University, Maastricht, The Netherlands. Participation was voluntary and no incentives, monetary, or otherwise, were offered in exchange for participation.

**Table 1:** Listings of motivations for participants for inhaling vapor from dried toad secretion containing 5-MeO-DMT

Understanding myself (42.9%)	
Solving problems (11,9%)	
All the above (2.4%)	
Other motivations (42.9%)	
*• Seeking something that I do not know*	
*• Heal myself, transform myself, accept myself, improve myself*	
*• Awakening*	
*• Medicine, new dose*	
*• To get back to myself, to heal, to be on good terms with myself*	
*• To get the experience I have been searching for*	
*• To heal my soul*	
*• To reconnect and professional potential in my work*	
*• My own research*	
*• To get closer to my core existence*	
*• Grow spiritually*	
*• To connect with all that is, transcendence, downloading universal knowledge and understanding of self*	
*• I want to learn how to support others*	
*• I want to love myself more. Understand the purpose of my life, my mission.*	
*• To reconnect*	
*• Whatever the medicine wants to show me in this moment.*	
*• Information for me and for the outside. New neuronal connections*	

### Setting

The sessions in the Czech Republic and The Netherlands were conducted in the open, e.g., in a garden or in nature at a secluded location, whereas the session in Spain was held inside in a tipi or a rented house (as these sessions took place in winter).

The goal of the sessions was to relieve individuals from psychological and physiological issues, increase their well-being, and facilitate personal insights/personal healing. The facilitator was often assisted by another person, either with a background in coaching (i.e., a person trained in supporting clients in achieving a specific goal through providing training and guidance), or a person with no (clinical) background in psychology or in psychedelic-assisted therapy at all. These individuals (with or without background) had previous experience with 5-MeO-DMT (if this was a toad, synthetic form, or plant was not specified), and got involved at the sessions out of personal interest. Participants on antidepressant medications or suffering from a mental health disorder such as schizophrenia, other psychoses, or cardiovascular illnesses were not allowed to participate in the sessions by the facilitator or the assistants. Yet it is important to highlight that many facilitators and their assistants lacked the clinical expertise to identify and exclude participants with contraindications.

The facilitator or their assistants provided participants with preparatory instructions prior to the session. Dietary preparation included not eating (red) meat and foods containing high levels of salt or sugar. Additional advice was to avoid stress, calm the mind, increase mindful introspection, focus on their intention, and abstain from alcohol and other substances to reduce purging and other adverse events that might arise from combining the use of multiple medications (polypharmacy) with the inhalation of vapor from dried toad secretion containing 5-MeO-DMT. In each session, a facilitator administered the vapor by placing the dried toad secretion in a glass pipe, and then heating it up using a torch lighter. During the administration of the vapor from dried toad secretion containing 5-MeO-DMT, the participant remained either standing or lying down on the ground or floor. The participant was instructed to inhale as much of the vapor as possible and to hold it in for some seconds before exhaling. The assistants remained on site after the session to assist in the integration process of the experience if this was deemed necessary and/or requested by the participant. The facilitator would chant (*Cantos de Haaco Camaac*, healing shamanic chants), and/or play various musical instruments (rattle, drums, flute) during the experience of the participants.

### Study procedure

The nature of the study was essentially observational and involved three consecutive assessments: a baseline assessment prior to inhalation of vapor from dried toad secretion containing 5-MeO-DMT, a second assessment conducted within 24 h post-session, and a follow-up assessment at 4 weeks after. Individuals who were at the location to inhale the vapor from dried toad secretion containing 5-MeO-DMT received a detailed explanation of the research aims and were invited to sign the informed consent in order to participate in the study. They completed a 30-min test battery consisting of questionnaires and a psychometric test prior to the session, which were used as baseline measures. The test battery was administered again on site after the acute effects of the vapor from dried toad secretion containing 5-MeO-DMT had disappeared, or within the following 24-h through an online survey created in Qualtrics. The follow-up assessment at 4 weeks after intake was administered online, also through Qualtrics.

### Samples

It is worth noting that none of the session facilitators weighed the dose of dried toad secretion that they administered in the glass pipe, but instead, they relied on visual inspection when preparing the individual dosages. We therefore do not know the actual doses of dried toad secretion that were given during sessions to each participant. Some facilitators reported having administered around 20–30 mg of dried toad secretion, while others reported to administer up to 100–120 mg. We did however obtain five samples of the dried *Bufo alvarius* toad secretion to identify the compounds that it contained. These samples were analyzed for the presence of the following tryptamines, *N*-methyltryptamine (NMT), *N*,*N*-dimethyltryptamine (DMT), 5-methoxy-*N*,*N*-dimethyltryptamine (5-MeO-DMT), 5-hydroxy-dimethyltryptamine (bufotenin, 5-HO-DMT), *N*,*N*-dimethyltryptamine (DET), 5-hydroxy-*Nω*-methyltryptamine (*N*-methylserotonin, NMe-5HT); steroid lactones (bufogenin, bufotalidin); and tryptophols (5-methoxytryptophol (5-MeO-tryptophol), 5-hydroxytryptophol (5-HO-tryptophol), and 5-methoxy-3-indoleacetic acid (5-MIAA)). The availability of different compounds in the toad secretion in milligrams (in case of tryptamines calculated to the weight of freebase) per 1 g of dried toad secretion in each of these samples is given in Table 2. For details of the analysis, see supplementary content in Appendix 1.

**Table 2:** Overview of compound concentrations in milligrams or micrograms per 1 g of secretion) in samples of dried secretion from the *Bufo alvarius* toad

Sample	5-MeO-DMT	Bufotenin	DMT	NMT	DET	NMe-5HT	Bufogenin	Bufotalidin	5-MeO-Tryptophol	5-HO-Tryptophol	5-MIAA
	[mg/g]	[mg/g]	[mg/g]	[μg/g]	[μg/g]	[mg/g]	[mg/g]	[mg/g]	[μg/g]	[μg/g]	[μg/g]
1	265.9	0.642	0.016	ND	< LOQ	0.028	< LOQ	< LOQ	0.696	ND	ND
2	203.6	0.600	0.032	ND	< LOQ	0.014	0.005	< LOQ	2.357	ND	ND
3	274.3	1.179	0.029	ND	< LOQ	0.050	0.013	< LOQ	1.307	ND	ND
4	283.3	3.530	0.022	ND	< LOQ	0.171	0.011	< LOQ	0.997	ND	ND
5	307.3	1.436	0.041	ND	0.415	0.064	0.008	< LOQ	3.053	ND	ND

*ND* not determined

*< LOQ* under the limit of quantification

### Test battery

The test battery consisted of six questionnaires; the Ego Dissolution Inventory (EDI), the 5-Dimensional Altered States of Consciousness Rating Scale (5D-ASC), the Satisfaction with Life Scale (SWL), the Depression, Anxiety, Stress Scale-21 (DASS-21), the Five Facets Mindfulness Questionnaire (FFMQ-15), and the Brief Symptom Inventory-18 (BSI-18). The BSI instrument overlaps with the DASS-21 but offers the additional sub-measure of somatization, which is why it was selected. In addition, a computerized version of the Picture Concept Test (PCT) was administered. It is worth noting that the EDI and 5D-ASC were administered only once and had to be filled out within 24 h after the session to measure the intensity of the acute effects of the inhalation of vapor from dried toad secretion containing 5-MeO-DMT.

### Ego Dissolution Inventory

EDI is an 8-item self-report scale that assesses the participant’s experience of ego dissolution (Nour et al. 2016). The participants answered the scale with making a mark on a line from either “No, not more than usually” (0%) to “Yes I experience this completely/entirely” (100%). The EDI is scored by calculating the mean percentage of all the 8 items. The higher the total score, the stronger the experience of ego dissolution. The internal consistency of the EDI is excellent with a Cronbach’s alpha (*α*) of 0.93. In this study, the original English version or non-validated, non-formal translations in Czech, Spanish, and Dutch of the EDI were used.

### 5-Dimensional Altered States of Consciousness Rating Scale

The 5D-ASC is a 94-item self-report scale that assesses the participants’ alterations from normal waking consciousness (Studerus et al. 2010). The participant is asked to make a vertical mark on the 10-cm line below each statement to rate to what extent the statements applied to their experience in retrospect from “No, not more than usually” to “Yes, more than usually.” The 5D-ASC contains the 11 subscales experience of unity, spiritual experience, blissful state, insightfulness, disembodiment, impaired control and cognition, anxiety, complex imagery, elementary imagery, audio-visual synesthesia, and changed meaning of percepts. In addition, we compiled the key dimensions of “oceanic boundlessness” which is one of the five key dimensions of the scale with a Cronbach’s alpha (*α*) of 0*.*95 that identifies mystical-type experiences and has been compared with the “heaven” aspect of Huxley’s mescaline account (Dittrich 1998). Information about the four remaining dimensions *anxious ego dissociation*, *visual restructuralization*, *auditory alterations*, and finally *reduction of vigilance* can be found in the referenced article. In this study, the original English version of the 5D-ASC was used in addition to non-validated, non-formal translation in Czech, Spanish, and Dutch made by our team. Internal consistencies across subscales in the Dutch, Czech and Spanish population were 0.94, 0.83, and 0.94, respectively.

The following questionnaires and computerized test were distributed at baseline, within 24 h, and at the 4-week follow-up.

### Satisfaction with Life Scale

SWL is a 5-item self-report scale (Diener et al. 1985). The purpose of the scale is to assess someone’s subjective satisfaction with life. The items are answered on a Likert scale ranging from 1 “Strongly disagree” to 7 “Strongly agree.” The total score is obtained by summarizing the points on each item. Scores can range from 5 to 35 points, indicating greater life satisfaction with higher scores. The scale has good psychometric properties. The original SWL in English has a Cronbach’s alpha (*α*) of 0.87, and was used in addition to a validated translation in Spanish with a Cronbach’s alpha (*α*) of 0.88 and a validated translation in Dutch with a Cronbach’s alpha (*α*) of 0.85 (Beuningen 2012; Vazquez et al. 2013). Finally, a non-validated translation of the questionnaire in Czech was obtained and used by the research team.

### Depression, Anxiety, and Stress Scale 21

DASS-21 is the shorter version of the original self-report questionnaire Depression, Anxiety, Stress Scale 42 (Henry and Crawford 2005). The purpose of this scale is to measure the constructs of depression, anxiety, and stress. The participants responded by rating the concordance with each statement from 0 “Did not apply to me at all” to 3 “Applied to me very much, or most of the time.” The sub-scale scores for depression, anxiety, and stress are calculated by summing the scores for the relevant items. The original DASS has 42 questions. To have the comparable scores, the sum of the DASS-21 is multiplied by 2. The total scale of the DASS-21 had a Cronbach’s alpha (*α*) of 0.93. In this study, the English version of the scale or the validated translations into Spanish, Czech, and Dutch were used. The Cronbach’s alpha (*α*) of the Spanish, Dutch, and Czech versions are 0.96, 0.95, and 0.87, respectively (Daza et al. 2002; Kučera et al. 2018; Wardenaar et al. 2017).

### Five Facets Mindfulness Questionnaire-15

This measure is a short form of the 39-item FFMQ (Baer et al. 2006). The FFMQ-15 is a self-report questionnaire which measures five different factors: (1) observe, noticing experience that are both internal and external such as thoughts and emotions; (2) describe: describing internal experiences; (3) acting with awareness: focus on the present activity; (4) non-judgment: not evaluating or judging the present experience; (5) non-reaction: allowing thoughts and feelings to come without acting or reacting upon them (Gu et al. 2016). The purpose of this scale is to obtain an understanding of an individual’s mindfulness-related capacities. The participants answered the FFMQ-15 by rating the concordance with each statement on a 5-point Likert scale that ranges from 1 “never true” to 5 “very often or always true.” The total FFMQ-15 score is obtained by adding all the sub-scale scores. The original scale has shown good internal consistency, and the Cronbach’s alpha (*α*) of each sub-scale was non-reaction = 0.77, non-judgment = 0.78, describe = 0.83, observe = 0.69, and awareness = 0.70(Baer et al. 2006). The original English version of the FFMQ-15 was used in addition to non-validated, non-formal translations in Spanish, Dutch, and Czech. Internal consistencies across subscales in the Dutch, Czech, and Spanish population were 0.66, 0.63 and 0.61, respectively.

### Brief Symptom Inventory 18

The Brief Symptom Inventory 18 (BSI-18) is a self-report scale which contains subscales on somatization, depression, and anxiety (Derogatis 2001). Participants were asked to rate a list of issues people can experience on a 5-point Likert scale ranging from 0 “None at all” to 4 “Extremely.” The BSI-18 is a reliable instrument for the assessment of psychological distress in both clinical and general populations with strong internal consistency and a Cronbach’s alpha (*α*) of each sub-scale from the validated English version was as follows: somatization = 0.82, depression = 0.87, anxiety = 0.84 (Franke et al. 2017). In this study, the original English version of the BSI-18 or the non-validated, non-formal translations in Spanish, Dutch, and Czech were used. Internal consistencies across subscales in the Dutch, Czech, and Spanish populations were 0.85, 0.85, and 0.83, respectively.

### Picture Concept Task

A creativity task with non-verbal stimuli was used, i.e., the PCT (Kuypers et al. 2016). The PCT was composed of stimuli from the Wechsler Preschool and Primary Scale of Intelligence and the Wechsler Intelligence Scale for Children. Each stimulus contains between 4 and 12 color pictures shown in two or three rows. The participants are instructed to find an association between one of the pictures of each row. They are asked to provide the correct solution as there is only one correct answer. The correct answers are taken as the dependent measure of convergent thinking. To assess divergent thinking, the participants were asked to provide as many alternative associations as possible by sticking to the rule: 1 item per row. This is the regular instruction included in the measures of divergent thinking, and it is used to calculate several parameters, i.e., originality, fluency, and the ratio of both, which reflect quantity and quality of divergent thinking. Fluency is defined as the number of alternative associations. Originality is calculated by evaluating the originality of the alternative association relative to those provided by all other participants in a session. Alternative answers were uniquely reported by a single participant received an originality score of 2. Answers that were shared with a single participant were valued as 1, and answers that were shared by 3 or more participants were rated as zero. Mean originality (creativity) scores and ratio originality scores, weighed for fluency (originality/fluency), were used as measures of divergent thinking. Three parallel versions of the PCT were used at baseline and the two follow-up measures after the session to avoid learning effects. Each parallel version consisting of 17 stimuli were shown, and participants had 30 s per stimulus.

## Statistics

A repeated-measure ANOVA using a linear mixed model analysis with *session* (3 levels) as the within-subject factor and *country* (3 levels) as the between-group factor was conducted. Fixed main effects included *session*, *country*, and *session* x *country*, using the maximum likelihood method. Covariance structure was chosen according to the best fit and included compound symmetry heterogeneous (CSH) as well as autoregressive (AR1) structures. Significant main effects of *session* and *session* x *country* were followed by separate contrasts between baseline and follow-up sessions with the Bonferroni adjustments for multiple comparisons. The alpha level of significance was set at 0.05. Pearson’s correlations were carried out to investigate the association between the level of ego dissolution and the experience of the altered states of consciousness during the acute effects induced by inhalation of the vapor from dried toad secretion containing 5-MeO-DMT and changes in outcome measures. Hedges’ *g* was calculated to estimate the effect sizes of significant mental health changes between sessions. The data was analyzed with the Statistical Package for the Social Sciences (SPSS).

## Results

Overall, 75 participants agreed to participate in the study at baseline. Of those, 42 participants completed the test battery both before inhalation of the vapor from dried toad secretion containing 5-MeO-DMT (baseline) and within 24 h, while only 24 completed the test battery at the third and final assessment 4 weeks after intake.

### Psychedelic experience

#### EDI

**Fig. 1:**
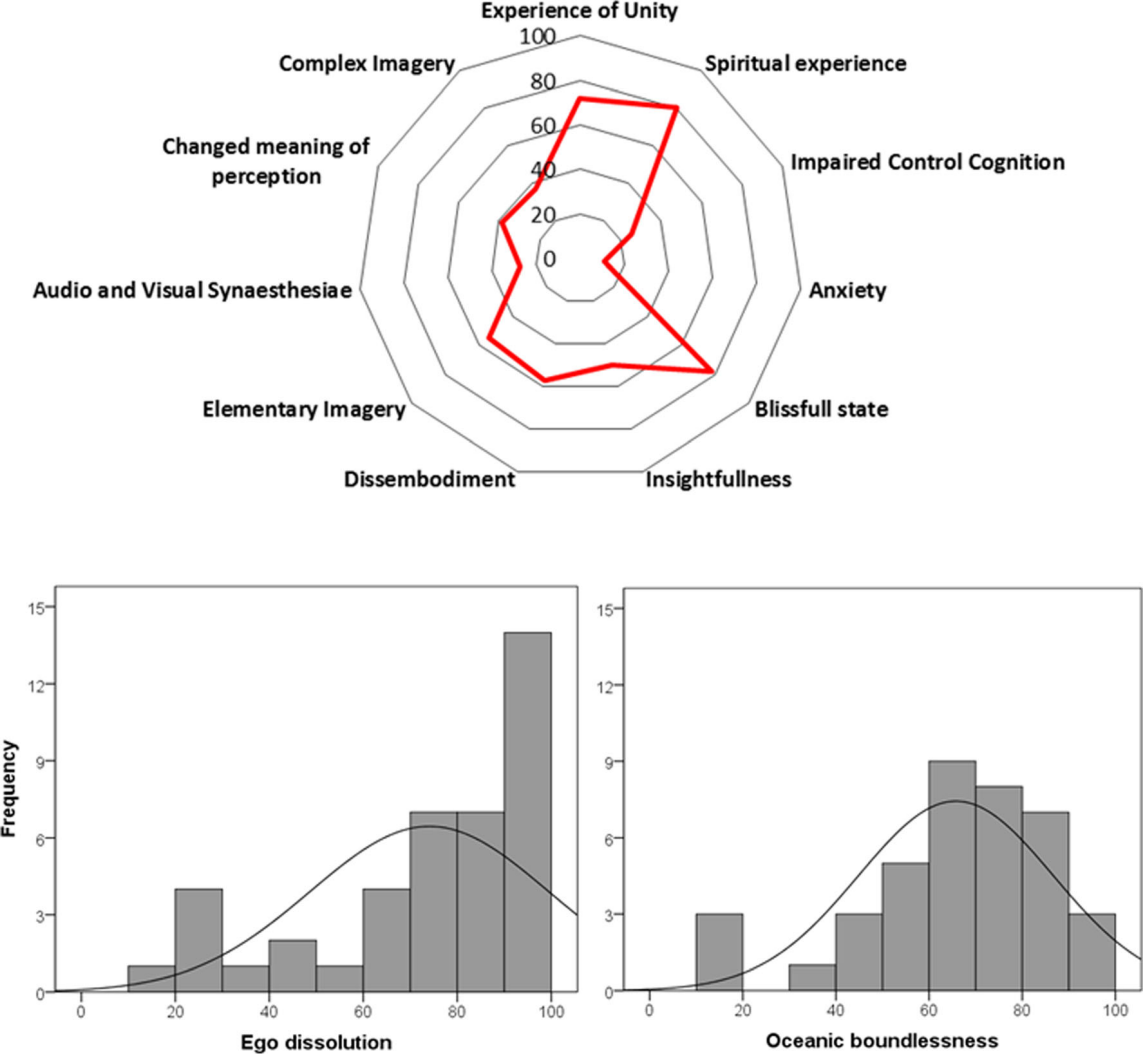
Mean ratings (range 0–100%) of the psychedelic experience as assessed with the 5D-ASC subscales after inhalation of vapor from dried toad secretion containing 5-MeO-DMT as well as frequency distributions of ego dissolution and oceanic boundlessness ratings (lower panels)

**Fig. 2:**
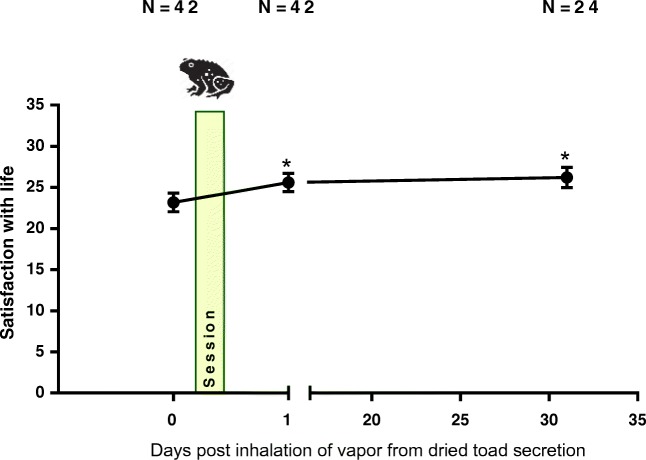
Mean (SE) satisfaction with life before and after inhalation of vapor from dried toad secretion containing 5-MeO-DMT (* = *p* < 0.05)

Mean (SE) ego dissolution rating was 74.24 (3.99). Overall, the total EDI rating varied between 0 (no dissolution) and 100 (maximal dissolution). About 20% of the sample rated their level of ego dissolution in the lower range between 0 and 60 on the EDI scale. The remainder of the sample achieved high levels of ego dissolution that ranged from 60 to 100 on the EDI scale. The distribution of EDI ratings is shown in Figs. 1 and 2.

### 5D-ASC

**Fig. 3:**
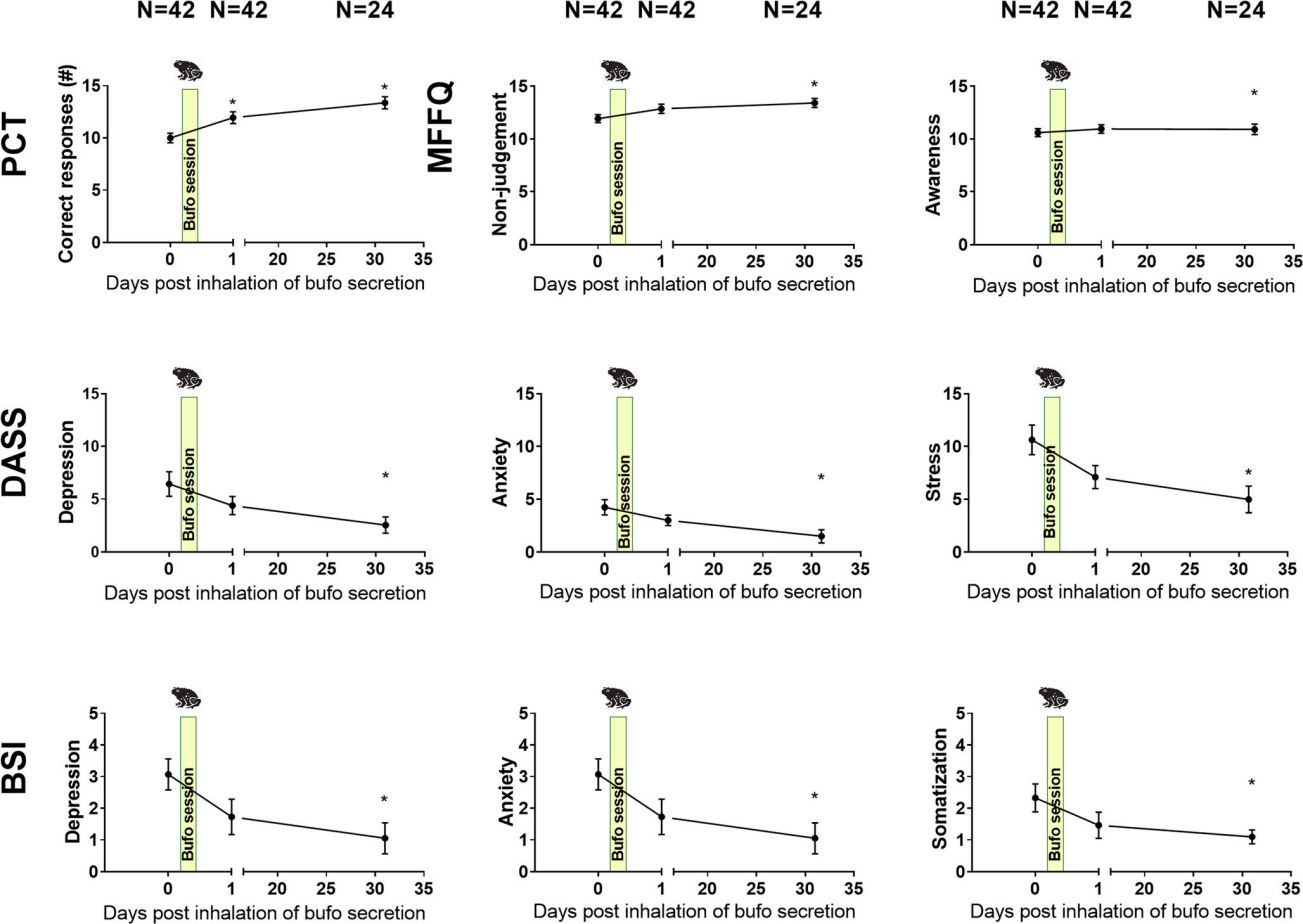
Mean (SE) correct responses (PCT), subjective ratings of mindfulness (FFMQ-15) depression, anxiety (DASS-21 and BSI-18), stress (DASS-21), and somatization (BSI-18) as a function of time after inhalation of vapor from dried toad secretion containing 5-MeO-DMT (* = *p* < 0.05)

**Fig. 4:**
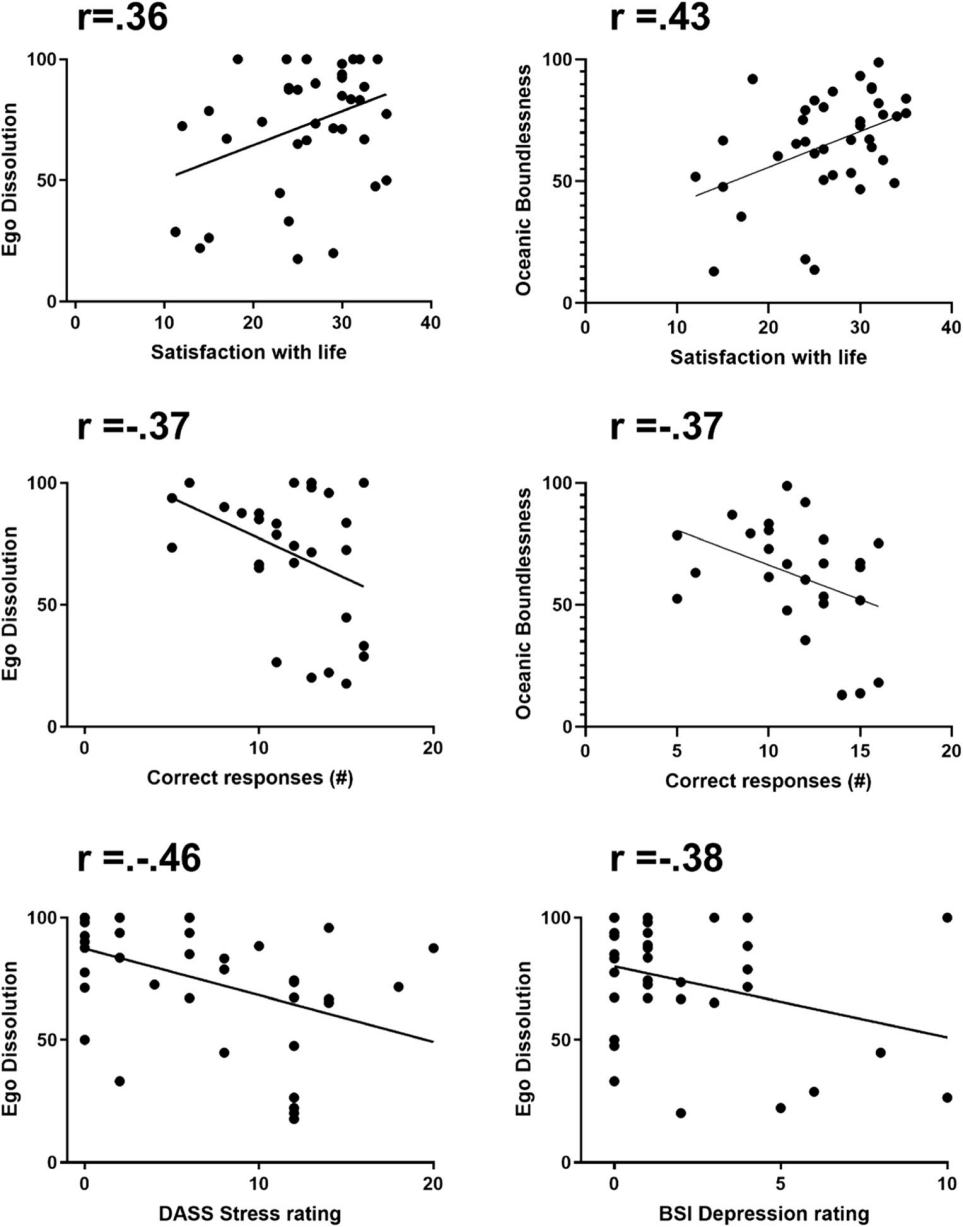
Pearson correlations between satisfaction with life and ego dissolution/oceanic boundlessness (upper panel), between correct responses in the PCT and ego dissolution/oceanic boundlessness (middle panel), and between ego dissolution and stress and depression ratings (lower panel) on the day after inhalation of vapor from dried toad secretion containing 5-MeO-DMT)

The mean rating of the psychedelic experience induced after inhalation of the vapor from dried toad secretion containing 5-MeO-DMT on the 11 subscales of the 5D-ASC is shown in Figs. 3 and 4. Rating of blissful state, spiritual experience, and experience of unity are the top three rated subscales. These subscales are also important contributors to the key-dimension oceanic boundlessness. The mean (SE) rating of oceanic boundlessness was 65.8% (3.35). About 30% of the sample rated their level of oceanic boundlessness in the lower range between 0 and 60%. The remainder of the sample achieved high levels of oceanic boundlessness that ranged from 60 to 100% on the 5D-ASC scale. Frequency distribution of the rating of oceanic boundlessness is shown in Fig. 1.

### Affect and cognition

#### SWL

Mixed model analyses revealed a main effect of *session* (*F*_2,44.12_ = 5.941; *p* = 0.005). Contrasts showed that satisfaction with life significantly increased within 24 h following the inhalation of the vapor from dried toad secretion containing 5-MeO-DMT (*p* = 0.022; Hedges’ *g* = 0.29), and persisted 4 weeks later (*p* = 0.012; Hedges’ *g* = 0.41), as compared with baseline. Mean (SE) satisfaction with life before and after inhalation of the vapor is shown in Fig. 2.

### DASS-21

Mixed model analysis revealed the main effects of *session* on depression (*F*_2,55.943_ = 4.348; *p* = 0.018), anxiety (*F*_2,55.033_ = 8.875; *p* < 0.001), and stress (*F*_2,51.874_ = 6.225; *p* = 0.004), the three subscales of the DASS-21. Separate contrast revealed that subjective ratings of depression, anxiety, and stress decreased within 24 h following the inhalation of the vapor from dried toad secretion containing 5-MeO-DMT, but failed to reach significance until 4 weeks later (*p* = 0.015, Hedges’ *g* = 0.79; *p* < 0.001, Hedges’ *g* = 0.69; *p* = 0.003, Hedges’ *g* = 0.66, respectively).

### FFMQ-15

The main effects of *session* reached significance on two mindfulness parameters: i.e., non-judgment (*F*_2,49.299_ = 5.27; *p* = 0.008) and awareness (*F*_2, 47.42_ = 3.28; *p* = 0.046). Separate contrasts revealed improvements in non-judgment (*p* = 0.009; Hedges’ *g* = 0.64) and awareness (*p* = 0.042; Hedges’ *g* = 0.12) 4 weeks after the inhalation of the vapor from dried toad secretion containing 5-MeO-DMT as compared with baseline. Sub-acute assessments of mindfulness however did not significantly differ from baseline. Furthermore, there were no significant differences between *session* or *countries* for the other FFMQ parameters, and interactions between *session* and *country* only reached significance on awareness (*F*_4,47.54_ = 4.63; *p* = 0.003). The interactions suggested that changes in awareness were higher in the Dutch and Spanish samples.

### BSI-18

Mixed model analyses revealed a main effect of *session* on depression (*F*_2,53.088_ = 4.014; *p* = 0.024), anxiety (*F*_2,46.778_ = 5.020; *p* = 0.011), and somatization (*F*_2,60.811_ = 4.640; *p* = 0.013). In addition, the analysis showed a main effect of *country* for somatization (*F*_2,37.134_ = 3.909; *p* = 0.029). Separate contrasts revealed that subjective ratings of depression, anxiety, and somatization decreased within 24 h of the inhalation of the vapor from dried toad secretion containing 5-MeO-DMT, but failed to reach significance until 4 weeks later (*p* = 0.023, Hedges’ *g* = 0.65; *p* = 0.009, Hedges’ *g* = 0.81; *p* = 0.011, Hedges’ *g* = 0.85, respectively). Furthermore, the changes in subjective ratings of somatizations were significantly higher in the Czech sample (*M* = 2.41, SE = 0.33) as compared with the Spanish sample (*M* = 0.73, SE = 0.50; *p* = 0.025), but not the Dutch sample. There was no interaction between *session* and *country*.

### Picture Concept Test

The mixed model analysis revealed a significant main effect of *session* (*F*_2,31.609_ = 5.34; *p* = 0.010) and *country* (*F*_2,31.76_ = 4.50; *p* = 0.019) on convergent thinking. Separate contrasts revealed that the number of correct solutions increased significantly after the inhalation of the vapor from dried toad secretion containing 5-MeO-DMT sub-acutely (*p* = 0.021; Hedges’ *g* = 0.66), and 4 weeks later (*p* = 0.002; Hedges’ *g* = 1.32), as compared with baseline. The mean (SE) convergent thinking scores were significantly higher (*p* = 0.003) in the Dutch 12.6 (0.5) and Czech sample 11.1 (0.6) as compared with the Spanish sample 9.4 (0.7). There was no interaction between *session* and *country*. Finally, none of the divergent thinking parameters were affected by any of the main factors or their interaction.

### Correlations

#### EDI

Sub-acutely following the inhalation of the vapor from dried toad secretion containing 5-MeO-DMT, EDI scores were positively correlated with changes in satisfaction with life as measured by the SWL (*r* = 0.357, *p* = 0.026), negatively correlated with DASS-21 ratings of stress (*r* = − 0.46; *p* = 0.003) depression as measured by BSI-18 (*r* = − 0.378, *p* = 0.016) and convergent thinking as assessed with the PCT (*r* = − 0.374; *p* = 0.046). EDI did not correlate with any changes measured at 4 weeks.

### 5D-ASC

Ratings of oceanic boundless were positively correlated to the rating of SWL (*r* = 0.43, *p* = 0.007) and negatively correlated with scores of convergent thinking (*r* = − 0.37; *p* = 0.046) during the day following the inhalation of the vapor from dried toad secretion containing 5-MeO-DMT. There were no correlations between ratings of oceanic boundlessness and rating of affect or cognitive measures at 4 weeks after the session.

## Discussion

The primary aim of the present study was to assess the sub-acute and long-term impact of the inhalation of vapor from dried toad secretion containing 5-MeO-DMT from the *Bufo alvarius* toad on psychological affect and cognition. A total of 42 participants completed (parts of) the test battery at baseline and after the session, while 24 participants completed the test battery 4 weeks after intake. Relative to baseline, ratings on satisfaction with life and convergent thinking significantly increased right after and over 4 subsequent weeks. Likewise, subjective ratings of non-judgment and awareness also increased over time and reached significance at 4 weeks. Ratings of depression, anxiety, and stress as assessed with DASS-21 or BSI-18 questionnaires decreased on the day after the session and reached significance at 4 weeks. Ratings of depression, stress, and convergent thinking on the day after the session were negatively correlated with levels of ego dissolution or oceanic boundlessness that were rated after the session. Scores on satisfaction with life the day after the session were positively correlated with scores on ego dissolution and oceanic boundlessness.

Inhalation of the vapor from dried toad secretion containing 5-MeO-DMT produced sub-acute and long-term improvements in subjective ratings of satisfaction with life, depression, anxiety, and stress. Relative to baseline, ratings of satisfaction with life significantly increased between 7 and 11% immediately after the session as well as 4 weeks after. Reductions in DASS ratings of depression (18%), anxiety (39%), and stress (27%) were evident right after the session and kept decreasing to 68%, 56%, and 48%, respectively, after 4 weeks, at which point the decrements reached statistical significance. BSI-18 ratings of depression, anxiety, and somatization indicated a similar pattern of improvement. Overall, this suggests that a single dose of vapor from dried toad secretion containing 5-MeO-DMT can bring about changes in affect and cognition that last for a prolonged period of time. Similar findings have been reported for 5-MeO-DMT and other related tryptamines. For example, in a recent survey, the use of 5-MeO-DMT in a naturalistic setting was associated with unintended improvement in depression and anxiety (Davis et al. 2019). Additionally, consumers of ayahuasca, a brew containing *N*,*N*-dimethyltryptamine (DMT), displayed significant reductions in ratings of depression and stress that persisted for 4 weeks after intake (Uthaug et al. 2018). Likewise, high-dose psilocybin produced large decreases in clinician- and self-rated measures of depressed mood and anxiety, along with increases in quality of life in cancer patients (Griffiths et al. 2016). The authors reported that at the 6-month follow-up, these changes were maintained, with about 80% of participants continuing to show clinically significant decreases in depressed mood and anxiety (Griffiths et al. 2016).

Notably, increased neurogenesis has been suggested as a central mechanism to underlie instant and long-term antidepressant properties of psychedelic compounds such as ketamine (Duman and Aghajanian 2012; Ma et al. 2017) and psilocybin (Catlow et al. 2013; Idell et al. 2017). Neurogenesis and synaptic plasticity may play a crucial role in how neural circuits regulate their excitability and connectivity and may link the neurobiology of depression to the therapeutic effects of glutamatergic drugs such as ketamine (Abdallah et al. 2015). In that respect, it is of interest that changes in glutamate transmission following ayahuasca use have been associated with improvements in a certain aspect of mindfulness as well (Sampedro et al. 2017). Alternatively, it has been proposed that therapeutic improvements in affect following the use of psychedelics might be mediated through sigma-1 receptor (S1R). The endogenous ligands for the S1R include neurosteroids and natural tryptamines, such as DMT and 5-MeO-DMT. Research suggests that both DMT and 5-MeO-DMT modulate innate and adaptive inflammatory responses through the sigma-1 receptor of dendritic cells (Szabo et al. 2014), which in turn might improve the etiology and symptomatology of neuropsychiatric diseases, such as depression.

Inhalation of vapor from dried toad secretion containing 5-MeO-DMT increased convergent thinking as assessed with the Picture Concept Task by 20 and 37% within 24 h and 4 weeks after the session. The PCT has previously been used in two observational ayahuasca studies to show that the brew increases divergent performance during the acute psychedelic state (Kuypers et al. 2016) but improves convergent thinking up until 4 weeks after the psychedelic experience (Uthaug et al., 2018). Likewise, the present data also suggests that the main impact of vapor from dried toad secretion containing 5-MeO-DMT on creativity is to improve convergent thinking following the psychedelic experience. However, in the present study, convergent thinking was also negatively correlated to subjective ratings of ego dissolution and oceanic boundlessness on the day after the session. This suggests that convergent thinking was poor in participants that reported a strong psychedelic experience and that the overall increase in convergent thinking relative to baseline may not be related to inhaling vapor but to another factor. For example, it cannot be overlooked that improvements in convergent thinking performance resulted from practice or learning effects that can arise from the repeated performance of the same task over time. We attempted to minimize learning effects by administrating parallel versions of the PCT at each time point, but the present study lacks a proper control group to evaluate the effectiveness of this precautionary measure. Alternatively, it can also not be overlooked that the overall improvement in convergent thinking after the session relates to improvements in mindfulness capabilities, such as acting with awareness, that contribute to optimization of cognitive functioning (Lebudaa et al. 2015). Long-term increases in convergent thinking may therefore coincide with improvements in mindfulness that were observed in the present sample of people inhaling vapor from dried toad secretion containing 5-MeO-DMT. In fact, inhalation of the vapor induced long-term improvements in two mindfulness parameters (e.g., non-judgment and awareness) as measured by the FFMQ-15. Similar findings have been reported in an observational study that compared aspects of mindfulness before and 24 h after an ayahuasca session using the long version of the FFMQ (Soler et al. 2016).

Moreover, most participants had a strong psychedelic experience as measured by the EDI and 5D-ASC and scored high on ratings of ego dissolution, as well as on the composite factor oceanic boundlessness. This is in line with previous findings about 5-MeO-DMT’s ability to induce strong mystical experiences (Barsuglia et al. 2018). Additionally, close inspection of the frequency distribution of ratings of ego dissolution and oceanic boundlessness demonstrated that 20–30% of the participants of the present study only had a low to medium psychedelic experience. This variability in psychedelic experience may have been caused by differences in doses administered at ceremonies, inhalation techniques, and the actual concentration of 5-MeO-DMT in the secretion from the *Bufo alvarius* toad used by different facilitators. Analysis of the five samples of dried secretion from the *Bufo alvarius* toad revealed that about 25–30% of the dried secretion consisted of the primary component 5-MeO-DMT. The samples also presented (very) low amounts of additional tryptamines such as bufotenine (0.08–0.18%), DMT, and *N*-methylserotonin (0.01–0.03%). Differences in tryptamine concentration observed in the dried toad secretion samples might very well affect the strength of the psychedelic experience, particularly when facilitators offer doses that span a wide range (i.e., 30–120 mg) as during sessions in the present study. Participants that inhaled vapor from a 30 mg dose of dried toad secretion may have received about 7.5–9 mg of 5-MeO-DMT whereas those who received a 120-mg dose of dried toad secretion may have inhaled up to 30–36 mg of 5-MeO-DMT. However, these estimates could also be lower as drug delivery may not have been complete due to individual differences in inhalation techniques, drug metabolism, and compound ratio following heating or when doses were only partially inhaled (Evans and Relling 1999; Hadidi et al. 1999; Shen et al. 2010; Yu et al. 2003). Nevertheless, it has become clear, from the visits to the session(s) where vapor from dried toad secretion containing 5-MeO-DMT was administered, that dose(s) are not standardized and vary between facilitators. This is likely one of the main reasons why the psychedelic experience of ego dissolution and oceanic boundlessness differed between the participants.

It could be argued that the changes in affect and cognition that were observed in the present study are not related to a pharmacological effect from the inhalation of vapor from dried toad secretion containing 5-MeO-DMT, but rather due to uncontrolled confounders such as expectations of participants prior to, and after, the session. It is conceivable that participants were psychologically distressed in anticipation of the session, which would explain why subjective stress as rated with the BSI-18 and DASS-21 was higher at baseline, prior to the session. Though a contributing role of expectation cannot be completely ruled out, there is also good evidence to suggest that changes in affect and cognition observed in the present study were related to the actual psychedelic experience. Correlational analysis demonstrated that ratings of satisfaction with life were positively correlated to levels of ego dissolution and oceanic boundlessness, whereas ratings of stress and depression were negatively correlated to the level of ego dissolution. This indicates that the magnitude of mental health changes that were observed after the session was directly associated with the strength of the actual psychedelic experience caused by inhalation of the vapor from dried toad secretion containing 5-MeO-DMT. This finding is in line with previous studies on psychedelics such as psilocybin and ayahuasca that have shown that stronger psychedelic experiences are associated with larger changes in therapeutic outcome measures (Bogenschutz et al. 2015; Griffiths et al. 2016; Roseman et al. 2017; Ross et al. 2016; Uthaug et al. 2018).

Several limitations of the present study should be taken into account, the main one being the lack of a proper control group. Thus, the present findings can therefore only be taken as a preliminary indication of the impact of inhaling vapor from dried toad secretion containing 5-MeO-DMT on mental health parameters. Placebo-controlled, randomized clinical trials are needed to replicate the current findings and to control for non-pharmacological factors that could explain the current findings as well. A second limitation is that about 40% (*N* = 33) of the volunteers that gave consent (*N* = 75) to participate in the current did not complete any measures after the ceremony. Of those that completed the assessments the day after the ceremony (*N* = 42), 50% did not complete the follow-up assessment at 4 weeks after the session. Their reasons for not completing the assessments are unknown but potentially could be driven by disappointments that emerged over the experience. Most participants listed either “understanding myself” or “solving problems” as their motivation for attending the sessions. Other motivations included self-development, the search for a spiritual experience or spiritual healing and curiosity. It is unknown whether the experience from inhaling vapor from dried toad secretion containing 5-MeO-DMT fulfilled the expectations and motivations of all participants. Mental challenges following a 5-MeO-DMT experience have been reported and can include feelings of grief, anxiety, panic, or paranoia (Barsuglia et al. 2017; Davis et al. 2018). Resurfacing of these experiences can occur even weeks after the experience (Sandoval 2006) and may lead to psychological difficulties, particularly in the absence of counseling (Johnson et al. 2008). The possibility thus exists that participants that did not fill out the follow-up measures did not do so because they experienced negative after-effects. In that case, their lack of responding would strongly bias the current findings. In this light, the fact that facilitators in the field often do not provide professional psychological counseling during and after the sessions is particularly worrisome. Structured counseling sessions during the acute psychedelic experience with additional sessions before and after would potentially increase the ability of users to integrate their experience with the vapor from dried toad secretion containing 5-MeO-DMT, increase our understanding of positive and negative outcomes of exposure to the vapor and reduce the number of volunteers lost to follow-up.

Additionally, as the use of vapor from dried toad secretion containing 5-MeO-DMT through means of inhalation increases, there are several ethical and ecological considerations worth highlighting. The International Union for Conservation of Nature (IUCN) red list of threatened species at present does not classify the *Bufo alvarius* toads as an endangered species. The classification dates back however to an assessment of the toad population in 2004 (Hammerson and Georgina 2004). The increasing popularity of inhalation of vapor at underground ceremonies and acknowledgement of its therapeutic potential may however affect the stability of the toad population in the long run. The increasing demand for the vapor will disturb the ecological equilibrium of the toads through the invasion of habitat, excessive milking, amphibian trafficking, and black-market dynamics (PsychedelicsToday 2018). Harassment of the *Bufo alvarius* toad however can be easily prevented by using synthetic 5-MeO-DMT instead of vapor from dried toad secretion containing 5-MeO-DMT. Switching to synthetic 5-MeO-DMT offers the advantage that it does not contain a cocktail of different compounds, can be produced and dosed in a standardized manner, and is therefore much safer to use in naturalistic as well as controlled research settings.

Despite the limitations of this study, the results underscore the effects of 5-MeO-DMT, the main active ingredient in the secretion from the *Bufo alvarius* toad, on mental health in humans. This study suggests that a single administration of vapor from toad secretion containing 5-MeO-DMT produces rapid and persistent improvements in satisfaction with life, mindfulness and psychopathological symptoms, and that these changes are associated to the strength of the psychedelic experience. These results provide evidence supporting further research examining the potential therapeutic effect of 5-MeO-DMT.

## Electronic supplementary material

ESM 1 (DOCX 31 kb)
