# Supplementary material for: A single inhalation of vapor from dried toad secretion containing 5-methoxy-N,N-dimethyltryptamine (5-MeO-DMT) in a naturalistic setting is related to sustained enhancement of satisfaction with life, mindfulness-related capacities, and a decrement of psychopathological symptoms
Source: Psychopharmacology (Berl). 2019 Apr 13;236(9):2653–66. doi: 10.1007/s00213-019-05236-w (PMC6695371; doi:10.1007/s00213-019-05236-w)
Supplement: Supplementary file 1 — (DOCX 31 kb) [file 213_2019_5236_MOESM1_ESM.docx]

Appendix 1

 Supplementary information about analysis of Bufo Alvarius Secretion

Standards of analytes were obtained from commercial suppliers (Sigma-Aldrich, CZ: 5-Methoxytryptophol (5-MeO-tryptophol), 5-Methoxy-3-indoleacetic acid (5-MIAA), 5-Hydroxy-*Nω*-methyltryptamine (*N*-Methylserotonin, NMe-5HT); BOC Sciences, USA: bufotalidin; Fluorochem Ltd, GB: 5-Hydroxytryptophol (5-HO-tryptophol); Biopurify Phytochemicals Ltd., CN: bufogenin) or syntesized at UCT Prague (*N*-Methyltryptamine (NMT), *N*,*N*-Dimethyltryptamine (DMT), 5-methoxy-*N*,*N*-dimethyltryptamine (5-MeO-DMT), 5-hydroxy-dimethyltryptamine (bufotenin, 5-HO-DMT), *N*,*N*-diethyltryptamine (DET)). Stock solutions of all the samples and analytes were prepared by dissolving the compounds in 50% MeOH/water (w/w, ρ=0.9156 g/mL @ 20°C) to a final concentration of 1 mg/mL using analytical balance KERN ABT ABT 220-5DNM with readout [d] = 0.01 mg. Working solutions were made by further dilution with the same solvent. For calibration curves mixtures of standards with nomial concentrations 1 pg/mL, 3 pg/mL, 10 pg/mL, 30 pg/mL, 10 pg/mL, 30 pg/mL, 1 ng/mL, 3 ng/mL, 10 ng/mL, 30 ng/mL, 10 ng/mL, 30 ng/mL, 1 μg/mL, 3 μg/mL, and 10 μg/mL respectively were prepared. Dilutions were made by pipetting but exact concentrations of analytes were calculated from weights measured using balances. The solutions were stored at -35°C.
 For UHPLC-MS analysis, an UltiMate 3000 LC system (Thermo, USA) consisting of a LPG-3400RS pump, a WPS-3000RS autosampler, and a TCC-3200RS column compartment was used. MS detection of the eluates from UHPLC system was carried out with a QTrap 6500 mass spectrometer (AB Sciex, Canada). The final optimized LC–MS method utilized a ZORBAX Eclipse Plus 95Å C18, 2.1 x 150 mm, 1.8 µm, 600 bar pressure limit (Agilent, USA; 959794-902) with ZORBAX SB-C18, 2.1 mm, 1.8 µm, UHPLC guard column (Agilent, USA; 821725-902). Mobile-phase A was water with 0.1% formic acid and 2mM ammonium formate, and mobile-phase B was pure methanol. The 0.1% formic acid served as a proton source for positive mode ionization in the electrospray ionization (ESI) source of the mass spectrometer. The gradient program was as follows: 0–0.1 min, 98% A; 0.1–8 min from 98% A to 2% A; 8–9.5 min, 2% A; 9.5–10 min from 2% A to 98% A; and equilibration of the column to 15 min. The autosampler temperature was kept at 4°C. The flow rate of the mobile phase was 200 μL/min, and the column temperature was adjusted to 30°C. The injection volume was 1 μL. For data acquisition and management, Analyst software version 1.63 and MultiQuant 3.0.3 were utilized (AB Sciex). A multiple reaction monitoring (MRM) MS method was developed for all studied analytes. Final MS conditions were: Turbo-V ion source equipped with ESI probe in a positive mode (ESI+), ion spray voltage 5350 V, curtain gas 20 psig, temperature 450°C, ion source gas 1 30 psig, ion source gas 2 30 psig.

Standards of analytes were obtained from commercial suppliers (Sigma-Aldrich, CZ: 5-Methoxytryptophol (5-MeO-tryptophol), 5-Methoxy-3-indoleacetic acid (5-MIAA), 5-Hydroxy-*Nω*-methyltryptamine (*N*-Methylserotonin, NMe-5HT); BOC Sciences, USA: bufotalidin; Fluorochem Ltd, GB: 5-Hydroxytryptophol (5-HO-tryptophol); Biopurify Phytochemicals Ltd., CN: bufogenin) or syntesized at UCT Prague (*N*-Methyltryptamine (NMT), *N*,*N*-Dimethyltryptamine (DMT), 5-methoxy-*N*,*N*-dimethyltryptamine (5-MeO-DMT), 5-hydroxy-dimethyltryptamine (bufotenin, 5-HO-DMT), *N*,*N*-diethyltryptamine (DET)). Stock solutions of all the samples and analytes were prepared by dissolving the compounds in 50% MeOH/water (w/w, ρ=0.9156 g/mL @ 20°C) to a final concentration of 1 mg/mL using analytical balance KERN ABT ABT 220-5DNM with readout [d] = 0.01 mg. Working solutions were made by further dilution with the same solvent. For calibration curves mixtures of standards with nomial concentrations 1 pg/mL, 3 pg/mL, 10 pg/mL, 30 pg/mL, 10 pg/mL, 30 pg/mL, 1 ng/mL, 3 ng/mL, 10 ng/mL, 30 ng/mL, 10 ng/mL, 30 ng/mL, 1 μg/mL, 3 μg/mL, and 10 μg/mL respectively were prepared. Dilutions were made by pipetting, but exact concentrations of analytes were calculated from weights measured using balances. The solutions were stored at -35°C.
 For UHPLC-MS analysis, an UltiMate 3000 LC system (Thermo, USA) consisting of a LPG-3400RS pump, a WPS-3000RS autosampler, and a TCC-3200RS column compartment was used. MS detection of the eluates from UHPLC system was carried out with a QTrap 6500 mass spectrometer (AB Sciex, Canada). The final optimized LC–MS method utilized a ZORBAX Eclipse Plus 95Å C18, 2.1 x 150 mm, 1.8 µm, 600 bar pressure limit (Agilent, USA; 959794-902) with ZORBAX SB-C18, 2.1 mm, 1.8 µm, UHPLC guard column (Agilent, USA; 821725-902). Mobile-phase A was water with 0.1% formic acid and 2mM ammonium formate, and mobile-phase B was pure methanol. The 0.1% formic acid served as a proton source for positive mode ionization in the electrospray ionization (ESI) source of the mass spectrometer. The gradient program was as follows: 0–0.1 min, 98% A; 0.1–8 min from 98% A to 2% A; 8–9.5 min, 2% A; 9.5–10 min from 2% A to 98% A; and equilibration of the column to 15 min. The autosampler temperature was kept at 4°C. The flow rate of the mobile phase was 200 μL/min, and the column temperature was adjusted to 30°C. The injection volume was 1 μL. For data acquisition and management, Analyst software version 1.63 and MultiQuant 3.0.3 were utilized (AB Sciex). A multiple reaction monitoring (MRM) MS method was developed for all studied analytes. Final MS conditions were: Turbo-V ion source equipped with ESI probe in a positive mode (ESI+), ion spray voltage 5350 V, curtain gas 20 psig, temperature 450°C, ion source gas 1 30 psig, ion source gas 2 30 psig.
